# Digital Microfluidics for the Detection of Selected Inorganic Ions in Aerosols

**DOI:** 10.3390/s20051281

**Published:** 2020-02-27

**Authors:** Shuquan Huang, Jessica Connolly, Andrei Khlystov, Richard B. Fair

**Affiliations:** 1 Department of Electrical and Computer Engineering, Duke University, Durham, NC 27708, USA; shuquan.huang@duke.edu; 2 Division of Atmospheric Sciences, Desert Research Institute, Reno, NV 89512, USA; jess.connolly@npl.co.uk (J.C.); andrey.khlystov@dri.edu (A.K.)

**Keywords:** digital microfluidics, aerosol impaction, droplets, sulfate sensing, ammonium sensing

## Abstract

A prototype aerosol detection system is presented that is designed to accurately and quickly measure the concentration of selected inorganic ions in the atmosphere. The aerosol detection system combines digital microfluidics technology, aerosol impaction and chemical detection integrated on the same chip. Target compounds are the major inorganic aerosol constituents: sulfate, nitrate and ammonium. The digital microfluidic system consists of top and bottom plates that sandwich a fluid layer. Nozzles for an inertial impactor are built into the top plate according to known, scaling principles. The deposited air particles are densely concentrated in well-defined deposits on the bottom plate containing droplet actuation electrodes of the chip in fixed areas. The aerosol collection efficiency for particles larger than 100 nm in diameter was higher than 95%. After a collection phase, deposits are dissolved into a scanning droplet. Due to a sub-microliter droplet size, the obtained extract is highly concentrated. Droplets then pass through an air/oil interface on chip for colorimetric analysis by spectrophotometry using optical fibers placed between the two plates of the chip. To create a standard curve for each analyte, six different concentrations of liquid standards were chosen for each assay and dispensed from on-chip reservoirs. The droplet mixing was completed in a few seconds and the final droplet was transported to the detection position as soon as the mixing was finished. Limits of detection (LOD) in the final droplet were determined to be 11 ppm for sulfate and 0.26 ppm for ammonium. For nitrate, it was impossible to get stable measurements. The LOD of the on-chip measurements for sulfate was close to that obtained by an off-chip method using a Tecan spectrometer. LOD of the on-chip method for ammonium was about five times larger than what was obtained with the off-chip method. For the current impactor collection air flow (1 L/min) and 1 h collection time, the converted LODs in air were: 0.275 μg/m^3^ for sulfate, 6.5 ng/m^3^ for ammonium, sufficient for most ambient air monitoring applications.

## 1. Introduction

Analysis of atmospheric aerosol content relies heavily upon the sensitivity of sampling and chemical analysis. Historically, aerosol concentrations were measured using filters that required several hours to collect sufficient mass of the target compounds to be detectable by the available analytical instruments. However, long sampling times and the nature of the filter-based media often lead to artifacts in aerosol sampling—gases can be adsorbed on the filter media or the collected particulate constituents could evaporate due to a pressure drop across the filter or due to changes in gas-phase concentrations during sampling [1,2,3,4]. The long sampling times also precluded researchers from detecting concentration changes that occurred on short time scales. These problems are compounded by relatively high labor costs, risks of contamination during handling, and inability to quickly assess the current aerosol concentration as filter extraction and analysis usually takes several days to process. Several on-line methods have been developed to avoid sampling artifacts and answer the need for automatic high time resolution measurements of aerosol chemical composition [5,6,7,8,9,10]. While these systems have been providing invaluable data in numerous laboratory and field studies, they have their disadvantages, such as fairly large form factors and high costs to purchase and operate. 

The use of microfluidic devices in atmospheric aerosol science has recently been reviewed [11,12]. While these reviews summarize selected microfluidic concepts, including the use of droplet technologies and MEMS-based fluidics, the point is made that microfluidic platforms have been under-utilized by the atmospheric aerosol community. Another important conclusion is that miniaturized devices for air quality assessment and airborne particle detection are receiving more attention in the face of substantially increased demand. 

The first report of impacting a microfluidic chip surface with aerosol and then scanning a microfluidic droplet electrically to collect the impacted particles for subsequent analysis was in 2004 [13]. Other examples of applying microfluidic devices to detect airborne aerosols include a MEMS-based air-microfluidic sensor employing a film-bulk acoustic resonator to directly measure the mass of the impinging particles [14]. It should be noted that measuring the particle mass requires single type particle collection of a known particle type. In addition, a MEMS-based hybrid microfluidic device was proposed in 2008 containing on-chip aerosol impaction and a micro-corona discharger to detect number concentration of the particles following particle charging [15]. It was reported that device performance was inadequate due to limited aerosol collection efficiency. One more recent example of a droplet-based aerosol collection and detection system used aerosol focusing directly into a collection microfluidic droplet containing assay reagents [16]. The presence of the particles was detected in the stationary droplet, so no droplet transport was attempted to a remote detection site. 

The goal of the present research was to find a simpler and cheaper solution that would provide comparable or improved time resolution and sensitivity efficiency to that of state-of-the-art aerosol sensing instruments. It was also a goal to be able to achieve high aerosol collection efficiency and particle-type selectivity in the presence of multiple-particle species aerosols. Towards this end, a compact Lab-on-Chip (LoC) aerosol sensing system is described that is designed to integrate and to automate the tasks of collection, preparation, extraction, and analytical detection of major inorganic ions in atmospheric aerosol on a single micro-fluidic platform. The instrument reported here is based on digital microfluidics technology that allows manipulation of micro-droplets by applying voltages to electrodes embedded into an insulating surface of the chip, i.e., without external pressure sources [17]. Micro-droplets can be transported, stored, mixed, reacted, or analyzed in a discrete manner using a standard set of basic instructions. The key feature of this technology is that it allows a seamless integration of aerosol collection and analysis on a single chip—after a brief collection phase, particles collected on the surface of the chip can be extracted by a moving droplet, which then can be analyzed for components of interest in a separate part of the chip. 

Target compounds are the major inorganic aerosol constituents: sulfate, nitrate, and ammonium. These compounds comprise the bulk of ambient water-soluble material that is the main determining factor of aerosol hygroscopicity [18,19]. Information on atmospheric concentrations of these components is essential for our understanding of both direct and indirect aerosol effects on climate. The small size, automation, time resolution, and the low detection limit of the microfluidic instrument will allow its use on research air balloons, unmanned aerial vehicles (UAV) and small aircraft, even in clean remote areas.

## 2. System Design

**Figure 1:**
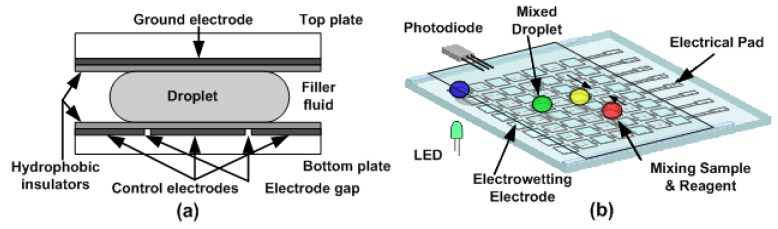
(**a**) Basic cell used in a digital microfluidic device; (**b**) Top view of microfluidic array.

The digital microfluidic system consists of top and bottom plates that sandwich a fluid layer, as shown in Figure 1. The bottom plate comprises conductive electrodes that are patterned on an insulating substrate and a dielectric layer that is deposited over the electrodes. The top plate is grounded during operation. Both surfaces of top and bottom plates are modified to be hydrophobic. When a voltage is applied to an electrode, the contact angle of the droplet on the dielectric surface is changed. Electric potentials applied between the electrodes and the top plate counter electrode reduce the contact angle of a droplet positioned on an electrode, thus increasing the wetting area of the droplet. Since changing the contact angle of liquid by an applied voltage can control the movement of liquid on a hydrophobic surface, a single droplet can be actuated across a dielectric covering two electrode plates with different voltages, and the droplet tends to move to the electrode with the higher applied voltage. The attraction is stronger when the applied voltage difference is larger, as is the droplet transport velocity. If these electrodes are arranged in a checkerboard pattern beneath a hydrophobic insulator, it is possible to direct the droplets of a specimen or reactant to a specific position for chemical reaction or analysis. In addition to its high efficiency and low energy consumption, this microfluidic operational platform with electrical controls has the potential for system miniaturization and integration with integrated circuit back-end control systems. 

**Figure 2:**
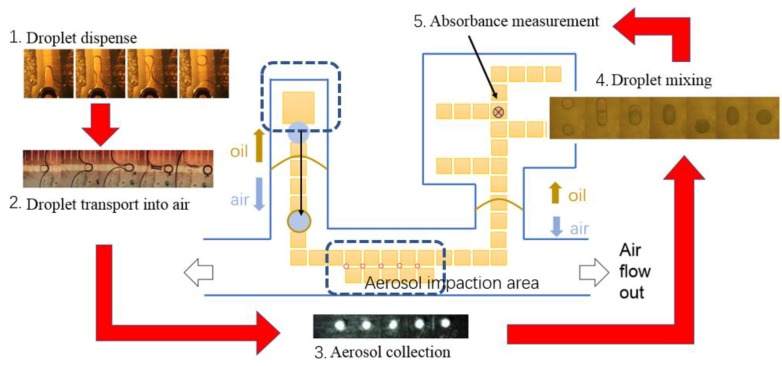
Diagram of inorganic ion concentration analysis process for aerosols. Middle: digital microfluidics chip layout. From left to right following the red arrows: the functional areas of the chip are displayed in sequence—droplet dispense, droplet transport, aerosol collection, mixing and absorbance detection by colorimetric testing.

The operation of the proposed integrated chip system is shown in Figure 2. The main idea behind the instrument is to collect aerosol on the surface of the digital microfluidics chip and to use electrowetting movement to dispense and transport a microliter droplet across the aerosol deposit, extracting water-soluble components. The droplet with the extracted material then will be mixed with a droplet (or droplets) of a compound-specific reagent to form a colored complex that will be photometrically detected on chip. Because all of these steps will be performed automatically, there is no need to remove the impaction substrate for analysis, and the sampling/analysis steps can be repeated numerous times.

The very small volume of the extraction droplet is beneficial for achieving low detection limits and/or short sampling times. This is demonstrated with the following analysis. To obtain a meaningful measurement, sampling needs to be done long enough to produce a detectable concentration of the analyte in the extraction droplet. The concentration in the extract relates to the air concentration and the sampling time through the following equation:

(1) $$C_{w}=\frac{C_{a}\;F_{a}\;t}{V_{w}}$$

in which *C_w_* is the concentration of the substance in the extract, *C_a_* is the concentration of the substance in air, *F_a_* is the sampling flow rate, *t* is the sampling time, and *V_w_* is the volume of the extraction droplet. The detection limit in air is then:

(2) $$LOD_{air}=\frac{LOD_{w}\;V_{w}}{F_{a}\;t}$$

in which *LOD_air_* is the detection limit in air, and *LOD_w_* is the limit of detection of the substance in the solution. 

From Equation (2) it follows that the *LOD* in air is proportional to the *LOD* of the analytical assay and the extraction volume, while inversely proportional to the sampling flow rate and sampling time. In the particle into liquid samplers (PILS) [5,8], the ratio *V*_w_/*F*_a_ is of the order of 0.01 mL of collected liquid per liter of air sampled. In the current design of our system (described in a separate section below), *F_a_* = 3 L/min and *V_w_* = 0.5 µL. Thus, the ratio *V_w_*/*F_a_* is a factor of 60 lower, i.e., better, than that in the PILS-type samplers. The detection limit of the on-chip colorimetric method, however, is about 50 times higher than that of the ion chromatographs used in PILS systems. The net result is that the *LOD_air_* of the current microchip design for sampling time of 1 min is comparable to that of PILS systems. 

The chip is divided into an oil-filled dispensing region, an air-filled aerosol collection region and an oil-filled reaction and colorimetric detection region. A challenge in implementing digital microfluidics for aerosol sampling was the requirement for the collection surface of the chip to be exposed to the sample air stream. In the common designs of digital microfluidic chips, the gap between the plates is filled with silicone oil. Silicone oil prevents evaporation of the droplets and also reduces the voltages required for transporting the droplets. Thus, we needed to (1) develop a mechanism of transporting a droplet from an oil-filled region of the chip into the collection area of the chip, which should not be filled with oil to allow sample air to access the collection surface, and (2) design a chip that allows aerosol collection between the top and bottom plates. Such a design is shown in Figure 2 where the location of the oil/air interfaces are indicated.

Aerosol impaction from an air stream is first performed on five sequential electrodes on the chip. After the air flow is stopped, a droplet of water is dispensed in an oil medium from a reservoir and moved through an oil/air interface by electrowetting to the impaction/collection area of the chip. The collected aerosol from the five sequential electrodes dissolves in the oil-clad buffer droplet, providing the five-fold concentration of aerosol in a single droplet. Next, the droplet with dissolved analytes is moved through an air/oil interface for chemical measurements and further analytical processing. All of the above steps can be completed in a cycle time on the order of few minutes.

### 2.1. Chip Design and Fabrication

The prototype microfluidic chip performs the functions of aerosol collection, extraction, and detection in times less than an hour. Further integration of impaction, collection and detection on chip to eliminate operator intervention should reduce throughput to minutes. All current functions are realized in dedicated sections on a digital microfluidic platform, shown in Figure 2. Different approaches for each function were first designed and tested individually, including aerosol impaction, droplet dispensing with different kinds of reagents, droplet transport between silicon oil/air interface and across the impaction area, and colorimetric assays for sulfate, nitrate, and ammonium measurement.

**Figure 3:**
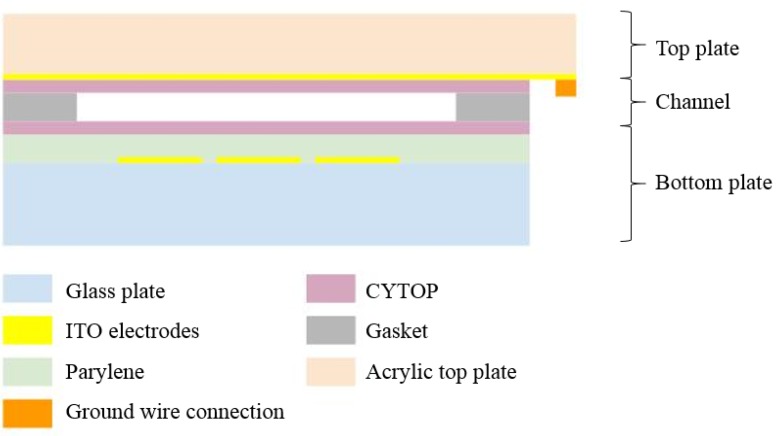
The cross-sectional view of the digital microfluidic chip.

The bottom plate and the top plate (Figure 3) of the microfluidic chip are fabricated separately, and later pressed together with a gasket layer put in between. The bottom substrate is on a 500 µm thick borosilicate glass wafer. 140 nm indium tin oxide (ITO) is sputtered and patterned into 0.7 mm × 0.7 mm electrodes with 0.05 mm separation space between each. Then a 1.3 μm thick Parylene C is deposited as the dielectric layer. Finally, 90 nm spin-on CYTOP^®^ (AGC Chemicals, Exton, PA, USA) covers the entire surface and the chip is baked at 100 °C followed by 190 °C, each for 10 min. This allows glass transition of CYTOP^®^ to happen. 

**Figure 4:**
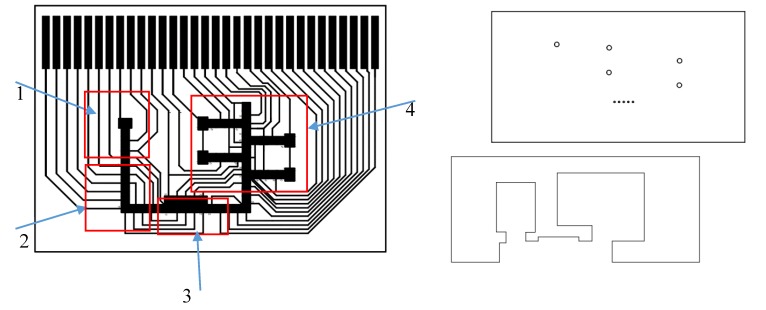
Left figure shows bottom plate, electrical interconnects and electrodes: Region 1: droplets are dispensed in oil from a reservoir in this area; Region 2: dispensed droplet are transported to impaction area and cross an oil/air interface; Region 3: aerosol impaction region in which droplets collected impacted particles in an air medium; Region 4: samples with collected particles cross air/oil interface and colorimetric analysis is performed. Top right: top plated with liquid loading holes and five impaction holes. Bottom right: outline of gasket spacer between top and bottom plates.

There were two design patterns for the electrodes. The first design (Figure 4) was on a 42 × 30 mm chip with 32 pins and 57 electrodes to perform the full function of collection and detection. Because the goal was to fit in more electrodes with fewer connection pads, each connection pad had to control multiple electrodes. Transportation electrodes were 1 mm × 1 mm squares. The five “T” shaped electrodes in Figure 4 are reservoirs, and the inlet holes were drilled on the top plate right above them. The aerosol analysis process happens on the chip from left to right: region 1: droplet dispensing; region 2, droplet transportation across the oil/air interface; region 3, aerosol deposition; region 4, colorimetric detection. The gasket is designed so that region 1 and region 4 are in ambient oil. The top plate has five drilled impaction holes right above the aerosol deposition electrodes. This chip design was used for aerosol deposition tests. A second chip design (28 × 23 mm) was used for colorimetric detection studies as described elsewhere [20].

### 2.2. On-Chip Aerosol Impactor

**Figure 5:**
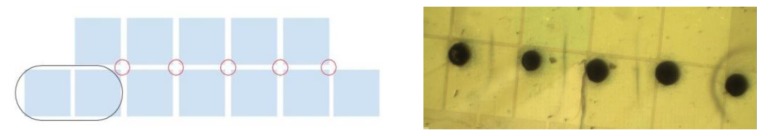
Left: The impaction area is composed of ten electrodes arranged in two rows. A droplet covers two electrodes. Right: Deposited aerosol particles on the CYTOP^®^ surface covering the actuation electrodes.

In general, aerosol collection requires a steady flow of air and a planar collecting surface. The digital microfluidics chip has a structure of a top ground plate and a bottom plate with electrodes. In the chip design here, the lower plate of the chip is used as a collecting plane, and the upper plate has five openings that are small enough to not affect droplet movement. The airflow comes through the openings to the channel of the chip, and after impacting on the lower plate, air flows out in a direction that is parallel to the lower plate. The impactor nozzle openings in the top plate allow particles to be deposited onto the CYTOP^®^ surface over the actuation electrodes. Figure 5 shows the position of the collection nozzles on the top plate and collection areas under the nozzles (left) and an ambient aerosol deposition pattern at the impaction area (right). Due to the impactor nozzles being offset from the electrodes, droplet pinning at impactor nozzles was avoided.

An inertial impactor was designed based on both empirical data and fluid mechanics following Marple’s rules [21]. Marple et al. have studied the collection efficiency of inertial impactors as a function of particle size and impactor geometry. The trajectory of a particle determines if the particle can be collected by the impaction plate. The solution for a particle trajectory is a function of the particle’s Stokes number. Larger Stokes number means that it is more difficult for the particle to change direction with the airflow. Thus, the collection efficiency is a function of Stokes number of the particle, Reynolds number of the airflow, and the physical dimensions of the impactor (nozzle diameter or nozzle width L, the distance between nozzle end and impaction plate S, and the nozzle length T). Experimental results show that the design considerations of an impactor require S/L = 1 for circular nozzles, S/L = 1.5 for rectangular nozzles, the Reynolds number should be between 500–3200, and T/L > 1, and the Stokes number should be 0.49 and 0.77 for round and rectangular nozzles respectively, Based on Marple’s impactor design criteria, the following impactor was integrated onto the microfluidic chip.

- Nozzle diameter: 0.24 mm (#88 drill), about 1/3 of actuation electrode size
- Number of nozzles: 5
- Total flow rate: 1.0 L/min
- Velocity at inlet nozzle: 131.0 m/s
- Reynolds number: 3132.7
- Pressure drop: about 0.10 atm
- Theoretical 50% efficiency cutoff diameter of aerosol, $d_{50}$: 162.7 nm

The target aerosol diameters were 0.1–1 μm, where most of the accumulation mode aerosol mass is found including that of sulfate, nitrate, and ammonium [18,22]. The cutoff diameter of 162.7 nm would allow most of the target particles to be collected. 

**Figure 6:**
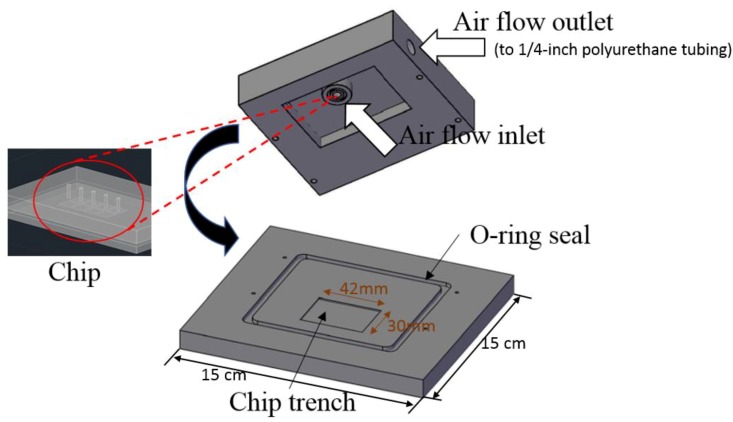
The design of on-chip inertial impactor and the chip-to-world chamber.

In order to evaluate the collection efficiency of the impactor design and to check the deposition pattern on the bottom plate, a chip-to-world chamber was designed, as shown in Figure 6. The impactor was tested using laboratory-generated ammonium sulfate aerosol. The aerosol was produced using a TSI constant output atomizer (Model3076, TSI Inc., Shoreview, MN, USA), dried and diluted with particle-free air. Aerosol size distributions were measured using a scanning particle sizer (SMPS 3936 with a CPC 3010, TSI Inc.). A comparison of size distributions measured before and after the chip provided the impactor cut-off characteristics. 

### 2.3. On-Chip Colorimetric Detection

As mentioned before, the research mainly focused on sulfate, nitrate and ammonium detection. For inorganic ions, the detection method is usually based on ion properties or wet chemistry. Ion exchange chromatography and capillary electrophoresis are solutions based on ion mobility and ion electric mobility. However, neither of these methods are suited for digital microfluidics. On the other hand, colorimetric analysis that involves formation of a colored complex with a target analyte and subsequent optical detection is suitable for digital microfluidic platforms. The benchtop colorimetric methods of detection for these ions are well established [23,24,25,26,27,28,29].

Two testing procedures were performed. In the first procedure, off-chip testing was performed that used the product solutions obtained in 1.5 mL tubes (analytes of six concentrations were used in each experiment, repeated several times). 200 μL of each solution was filled into a well plate and the absorbance was measured with a plate reader (Tecan Infinite 200 Pro, Tecan Group Ltd., Mannedorf Switzerland). In the other procedure, on-chip tests for the three analytes were performed, and the results were compared with the Tecan plate reader results. Because the volume of the solution on the chip was only a few microliters, much smaller than the volume of the off-chip test, and the optical path length, which equals to the channel height, was short, and because the on-chip mixing step involved a 1:1 volume ratio, the solution concentrations used by the on-chip test are slightly adjusted.

**Figure 7:**
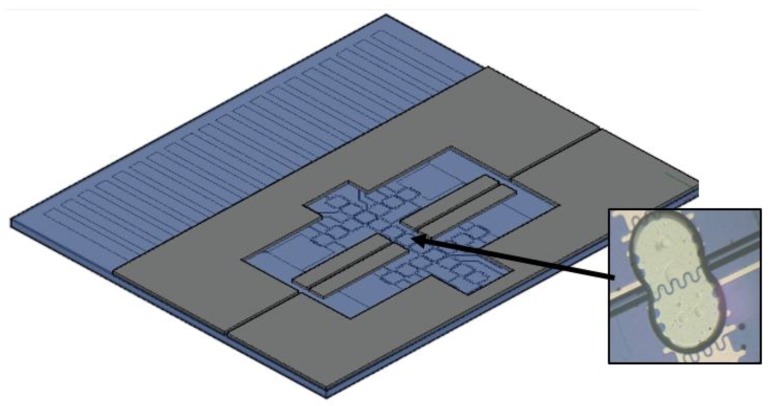
Channel made from SU8 to fix the optical fibers in place and a droplet held by two activated electrodes between horizontal optical fibers.

For on-chip detection, two bare optical fibers were fixed horizontally inside the chip between the top and bottom plates. To keep the optical fibers in place, a step was added before dielectric deposition to build an SU-8 channel of ~150 μm (channel height was 240 μm), as shown in Figure 7. The optical fibers were carefully cleaved to create a flat end surface and were coated with a hydrophobic layer, so that droplets could easily pass between the fibers without being pinned. The other ends of these two optical fibers were connected to the LED source and the spectrometer (Ocean Optics USB2000, Largo, FL, USA). Details on the measurement approach and reproducibility are presented elsewhere [14]. Absorption wavelengths used were 540 nm for detecting nitrate, 365 nm for ammonium, and 608 nm for sulfate.

## 3. Results

### 3.1. Aerosol Collection Efficiency

To test the impactor collection efficiency, laboratory-generated ammonium sulfate aerosol was used. The collected aerosol particles formed separate deposits directly under the five top plate openings. These deposits were distributed with equal distances across six electrodes. The collection efficiency at different sizes was calculated by measuring the ratio of size-dependent aerosol concentrations in upstream (before on-chip impaction) and downstream airflows (after on-chip impaction).

**Figure 8:**
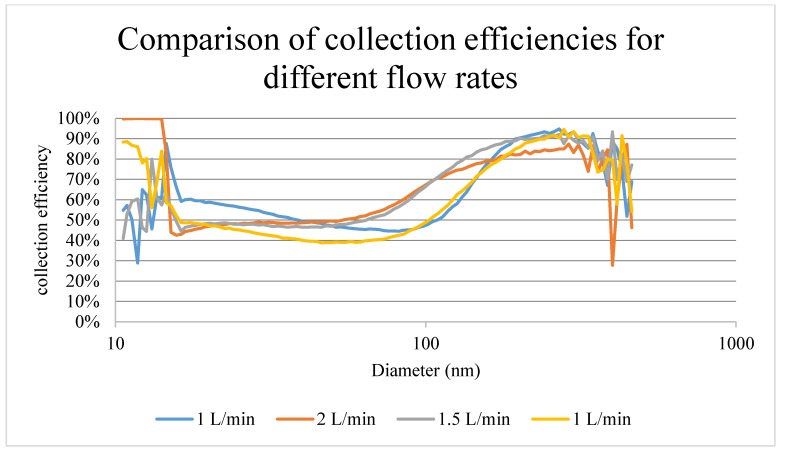
Collection efficiency as a function of particle diameter for three different flow rates and a repeat measurement at 1 L/min.

Three different flow rates were tested: 1 L/min, 1.5 L/min and 2 L/min, and 1 L/min was repeated. For every test a new chip was used. As show in Figure 8 there was not a significant difference in collection efficiency. The cutoff size for 50% efficiency was less than 100 nm (66.1 nm and 55.2 nm) for flow rates greater than 1 L/min and slightly larger than 100 nm (105.5 nm and 109.4 nm) for a flow rate of 1 L/min. This cutoff size agrees with theoretical calculations, and 1 L/min was a suitable flow rate for the system. The observed collection efficiency differed from that of classical impactors in that it did not decrease to 0% at smaller sizes. This deviation is explained by the small dimensions of the chip that promote diffusional deposition of particles smaller than 100 nm in diameter. The observed collection efficiency demonstrates that the impactor was capable of collecting accumulation mode particles where most of the fine ambient aerosol was found. It should be noted that the pressure drop at these flow rates was fairly low, 0.037 atm, which allows operating the chip collector with a small pump.

In addition to collecting particles in the size range of interest, the system needed to quantitatively recover the collected material. After the collection was completed, a droplet was created from the dispensing reservoir and moved to the collection plane. This droplet then performed two scans across the entire impaction area, which completely dissolved the deposited aerosols. 

### 3.2. Multianalyte Colorimetric Detection Results

#### 3.2.1. Sulfate and Ammonium

**Figure 9:**
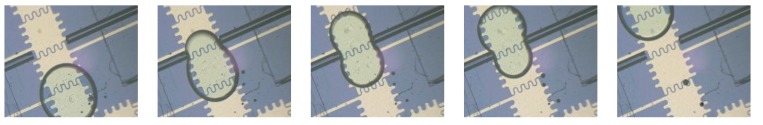
The movement of an MTB-Ba sulfate solution droplet during a measuring.

**Figure 10:**
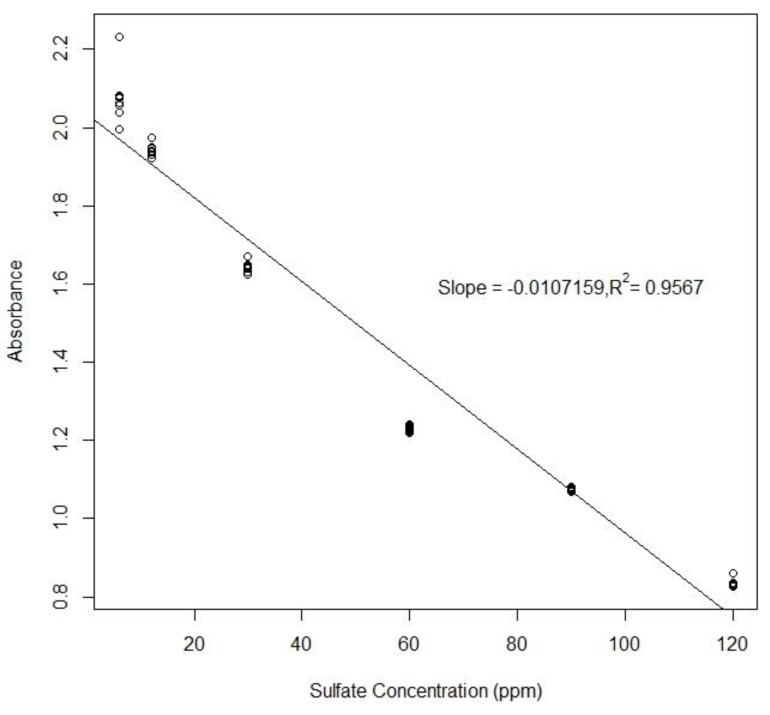
The absorbance of 12 measurements (averaged over measuring periods) for six different concentrations of sulfate.

**Figure 11:**
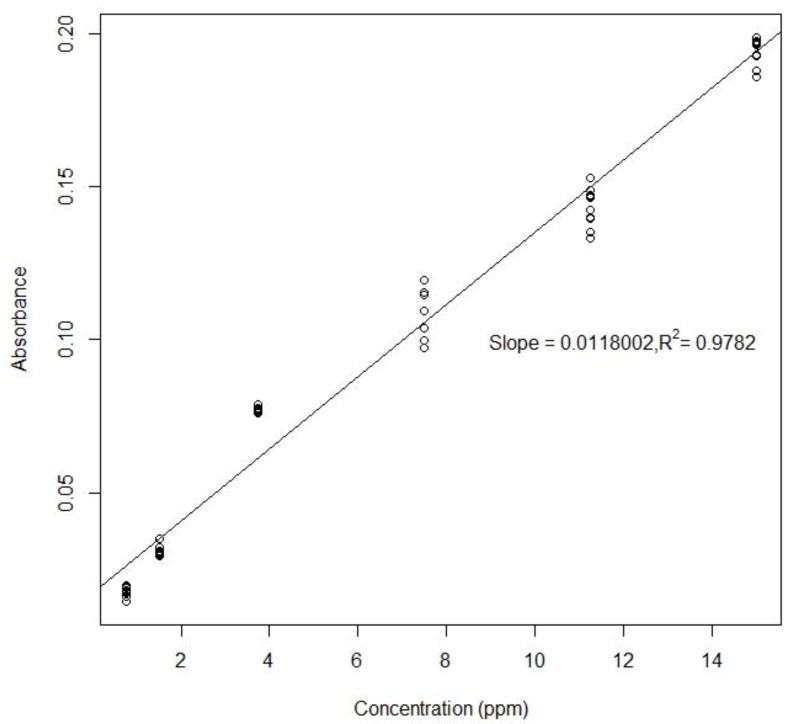
Measured absorbance (averaged over measuring periods) for six concentrations of ammonium.

To create a standard curve for each analyte, six different concentrations of standards were chosen for each assay and sequentially dispensed from on-chip reservoirs. The droplet mixing between sample and reagent was completed in a few seconds and the final droplet was transported to the detection position as soon as the mixing was finished. The droplet was moved back and forth through the gap between two optical fibers for multiple times. The droplet was maintained between two electrodes when measurement was taken (Figure 9). By turning on individual electrodes, it was easy to adjust the droplet position relative to the optical fibers. The results of the colorimetric measurements for sulfate and ammonium are shown in Figure 10 and Figure 11. 

The methylthymol blue (MTB) assay is based on competition between MTB and sulfate for barium ions [23]. As sulfate concentration increases, light absorption due to the MTB-barium complex decreases resulting in a negative relationship between the sulfate concentration and the absorbance (Figure 10). To find the limit of detection of sulfate, the lowest concentration of light absorbing analyte that would cause the smallest absorbance value, or the highest concentrations of sulfate (120 ppm), was used. Measurements were taken for five mixed droplets, each with eight points. The results show that the standard deviation of all 40 points was 0.041, corresponding to a limit of detection to create a standard curve for each analyte. We chose six different concentrations of standards for each assay. The droplet mixing was completed in a few seconds and the final droplet was transported to the detection position as soon as the mixing was finished.

The limit of detection test of ammonium was performed by using standards with the lowest concentration, 0.75 ppm. The result shows that the standard deviation of all 40 points was 0.0066, corresponding to 0.256 ppm for the limit of detection of ammonium.

#### 3.2.2. Nitrate

For nitrate, it was impossible to get stable measurements. During the final mixing step of the analyte with reagent droplets, dark spots in the droplet were observed, likely due to formation of a precipitate. These dark spots appeared to be suspended in the droplet and flowed around with the current inside the droplet. The number of dark spots increased significantly during the first three or four minutes after mixing, then became stable. Although these dark spots were not observed during a bench-top plate reader test, they did not disappear when the electrowetting voltage was turned off. Gas bubbles were also observed, but their origin is unclear. The absorbance values slightly increased during the first three or four minutes. The reason for precipitation is yet unknown, but the hypothesis is that the acidic reagent in the nitrate assay attacks the coating material of the top plate in the presence of applied voltage.

**Figure 12:**
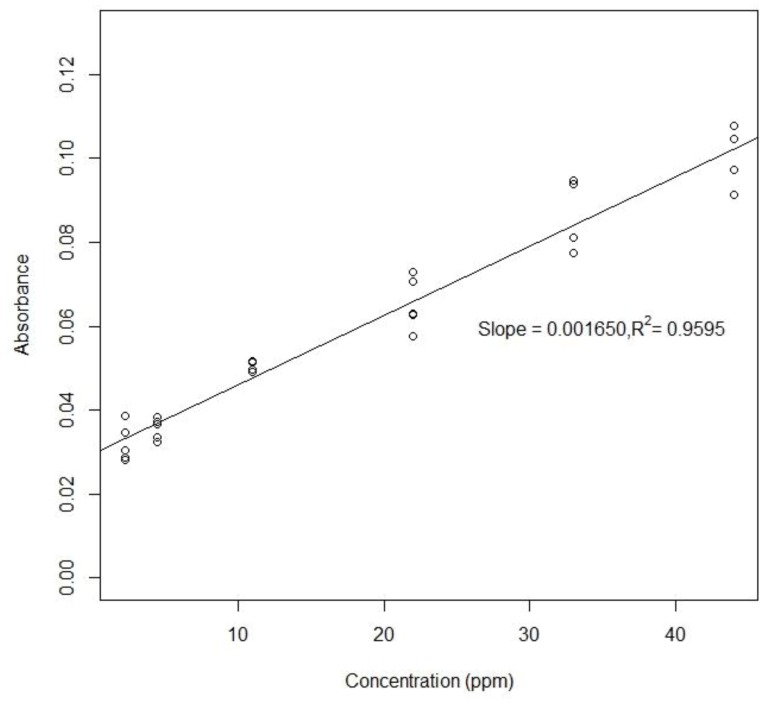
Five consecutive measurements after the absorbance became stable were recorded for each of the six nitrate concentrations.

If five relatively more stable measurements at 150 s after mixing are taken, the absorbance and concentration do have the linear relationship (Figure 12). However, the linear fitting curve has a nonzero y-intercept, which means the precipitation phenomenon undermined the sensitivity of this nitrate detection method.

### 3.3. Comparison between Off-Chip Results and On-Chip Results

Table 1 is a comparison between the performance of a Tecan spectrometer and a digital microfluidics chip system on all three colorimetric assays. In general, off-chip (manual process using plate reader) and on-chip methods generated comparable results, which are reflected in the following aspects: (1) All $R^{2}$ values from the data plots were around 0.95, indicating that the on-chip method could reproduce the linear relationship exhibited in the off-chip method. (2) The LOD of the on-chip measurement method for sulfate was close to that obtained by the off-chip method. The LOD of the on-chip method for ammonium was about five times larger than the off-chip method. This is probably because for sulfate, the range of linear changes in absorbance was larger, between 0.8–2.1, while for ammonium the absorbance was only 0.02–0.2. Further experiments on the adjustment of analyte or reagent concentrations may improve the on-chip LOD. 

Even though the on-chip LOD was larger, for the current impactor design characteristics (collection air flow of 1 L/min) and 1 h collection time the converted LOD in air were: 0.275 μg/m^3^ for sulfate, 6.5 ng/m^3^ for ammonium, sufficient for most ambient air monitoring applications [18]. Based on the results in Table 1, on-chip colorimetric assays can obtain measurements comparable to off-chip methods and can be applied to ion detection in air. 

Finally, in the field, impacted aerosol in air will contain multiple species of particles. Previous studies [28,29] have been performed on absorbance interferences from three coexisting ions: sulfate, nitrate and ammonium using colorimetric measurements. These interference measurements were repeated in our off-chip plate reader. The effect of interfering ions on the concentration measurement of any specific ion was found to be negligible over the ranges studied.

## 4. Conclusions

An aerosol detection system that combines digital microfluidics technology and aerosol impaction and chemical detection on the same chip was designed and tested. The system is small and fast, and can perform measurements combined with chemical detection in times less than an hour. Further integration of impaction, collection and detection on chip to eliminate operator intervention should reduce throughput to minutes. Aerosol impaction is accomplished using an inertial impactor built into the chip by scaling dimensions while keeping Reynolds number in an optimal range. The deposited air particles were densely concentrated on the bottom plate of the chip in a fixed area and formed clear patterns. The measured collection efficiency was larger than 50% for particles in the size range of 100 nm increasing to about 95% for 500 nm particles. Similar efficiencies are expected for particles up to 1 µm. The on-chip analytical assays exhibited limit of detection and the range of linear response similar to those determined using benchtop tests. 

The prototype of the chip has been fabricated and preliminary results on impaction and colorimetric methods have proven the feasibility of high-throughput on-chip detection of inorganic ions in aerosol. To create a standard curve for each analyte, six different concentrations of liquid standards were chosen for each assay and dispensed from on-chip reservoirs. The droplet mixing was completed in a few seconds and the final droplet was transported to the detection position as soon as the mixing was finished. Limits of detection (LOD) were determined to be 11 ppm for sulfate and 0.256 ppm for ammonium. For nitrate, it was impossible to get stable measurements. The LOD of the on-chip measurements for sulfate was close to that obtained by the off-chip method using a Tecan spectrometer. LOD of the on-chip method for ammonium was about five times larger than the off-chip method.

For the current impactor collection air flow of 1 L/min and 1 h collection time the converted LOD in air were: 0.275 μg/m^3^ for sulfate, 6.5 ng/m^3^ for ammonium, which are sufficient for most ambient air monitoring applications [18]. No significant interference from other ions that could be found in ambient aerosols was detected over the studied concentration ranges. 

## Figures and Tables

**Table 1:** Comparison of the performance of Tecan spectrometer and digital microfluidics chip system on all three colorimetric assays.

		Off-Chip (Plate Reader)	On-Chip (Horizontal)
sulfate	slope	0.0026	0.0107
	R2	0.9514	0.957
	absorbance range	1.35–1.65	0.8–2.1
	limit of detection	7 ppm	11 ppm
	CV	0.2–4%	0.3–2.7%
nitrate	slope	0.0036	0.00165
	R2	0.9961	0.9595
	absorbance range	0.04–0.18	0.02–0.1
	limit of detection	0.11 ppm	N/A
	CV	1.5–5%	N/A
ammonium	slope	0.1503	0.0118
	R2	0.9645	0.9782
	absorbance range	0.1–2.3	0.02–0.2
	limit of detection	0.0565 ppm	0.256 ppm
	CV	7–14%	0.4–11.6%
